# The value of the cinematic volume rendering technique: magnetic resonance imaging in diagnosing tumors associated with the brachial plexus

**DOI:** 10.1186/s40001-023-01416-9

**Published:** 2023-12-06

**Authors:** Rui Chen, Yuncai Ran, Yanglei Wu, Haowen Xu, Junxia Niu, Yong Zhang, Jingliang Cheng

**Affiliations:** 1 Department of Magnetic Resonance Imaging, The First Affiliated Hospital of Zhengzhou University, Zhengzhou, China; 2 Department of Interventional Neuroradiology, The First Affiliated Hospital of Zhengzhou University, Zhengzhou, China; 3 MR Collaborations, Siemens Healthineers Ltd, Beijing, China

**Keywords:** Brachial plexus nerve, Tumor, Blood vessels, Cinematic volume rendering technique (cVRT)

## Abstract

**Purpose:**

To examine the diagnostic advantages and clinical application value of the cinematic volume rendering technique (cVRT) when evaluating the relationship between the brachial plexus, peripheral tumor lesions, and blood vessels.

**Materials and methods:**

Seventy-nine patients with brachial plexus tumors between November 2012 and July 2022 were enrolled in our study. All patients underwent T1WI, T2WI, three-dimensional short recovery time reversal recovery fast spin-echo imaging (3D-STIR-SPACE), and the T1WI enhancement sequence. In addition, cVRT was used to render and obtain a three-dimensional model that clearly showed the location and tissue structure of the brachial plexus nerves and the tumor in all directions.

**Results:**

Seventy-one patients (mean age, 47.1 years; 33 males, 38 females) with tumors around the brachial plexus were included in the study. The brachial plexus nerve, surrounding tumor lesions, and vascular anatomy of all patients were well displayed with cVRT. The tumors of 37 patients manifested as unilateral or bilateral growths along the brachial plexus nerve and were fusiform, spherical, or multiple beaded; seven patients' tumors pushed against the brachial plexus nerve and were circular, lobular, or irregular; sixteen patients' tumors encircled the brachial plexus nerve and were spherical; and eleven patients' tumors infiltrated the brachial plexus nerve and had irregular morphology. The mass has a moderately uniform or uneven signal on T1WI and a high or mixed signal on T2WI. After enhancement, the signal was evenly or unevenly strengthened.

**Conclusions:**

cVRT clearly showed the origin of tumors associated with the brachial plexus and their relationship with the nerves and peripheral blood vessels, providing reliable information for clinical diagnosis and treatment.

## Introduction

The brachial plexus is a complex anatomical structure that provides innervation to the upper limbs, shoulders, and upper chest. Injury of the brachial plexus will seriously affect the patient's limb and may lead to partial or complete loss of upper limb function and even lifelong disability [1]. The brachial plexus nerve may be infiltrated by surrounding neoplastic lesions, which affects its function. In upper limb tumors, the incidence of tumors in the brachial plexus nerve area is approximately 1–4.9% [2, 3], of which benign tumors account for 76.9–91.6% [4, 5]. Surgical resection is the most effective method of treating tumors around the brachial plexus. The patient's clinical treatment method closely depends on the size, growth site, and biological behavior of the tumor. Therefore, the positioning and qualitative diagnosis of the tumor before surgery are critical for clinicians.

Magnetic resonance imaging possesses the characteristics of high-tissue contrast. Therefore, it is the best noninvasive examination method for diagnosing brachial plexus malignancy [6–8]. In recent years, with the widespread application of magnetic resonance brachial plexus neuroimaging, the imaging research of brachial plexus-related tumors has been receiving more attention. Brachial plexus neuroimaging can accurately describe the imaging characteristics of tumors associated with the brachial plexus, including the lesion size, location, source, and surrounding tissue involvement, to guide surgical methods and evaluate resection ability [9].

The 3D-STIR-SPACE sequence achieves displaying the brachial plexus well by inhibiting background fat. At the same time, this sequence can demonstrate the location, origin, and scope of tumors associated with the brachial plexus. It can be reconstructed in three dimensions through post-processing to clearly show the spatial positional relationship between the tumor and the brachial plexus. However, the 3D-STIR-SPACE sequence has certain limitations in showing the encircling and infiltration of the brachial plexus by tumors near it. The cinematic volume rendering technique (cVRT) builds on the principles of Volume Rendering (VR), which involves integrating high-resolution volumetric data from medical images to create a 3D representation of the internal structure of an object [10]. In this study, we aimed to use cVRT to fuse the brachial plexus with surrounding tumor lesions and vascular anatomy in three dimensions to demonstrate better the relationship between tumors associated with the brachial plexus and the plexus itself. In addition, we aimed to provide more intuitive and accurate image information for clinical use.

## Materials and methods

### Study participants

**Fig. 1:**
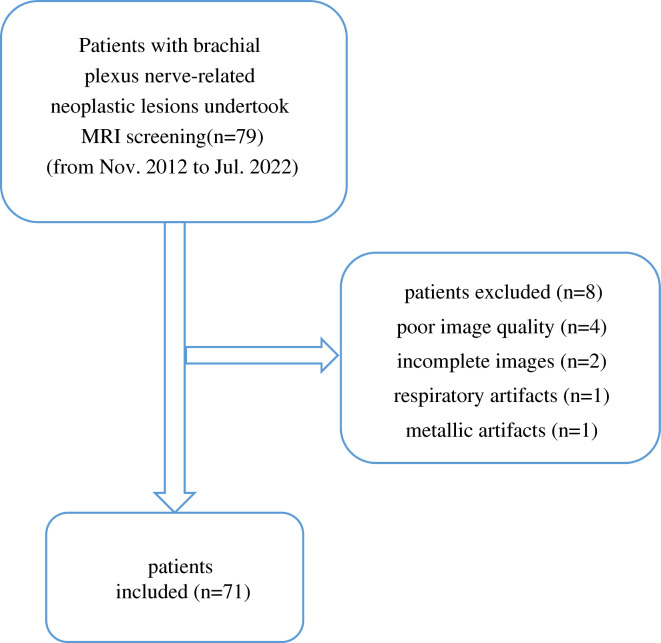
Participant selection flowchart

Seventy-nine patients with brachial plexus nerve-related neoplastic lesions were analyzed from November 2012 to July 2022 at our institution. Eight of these patients were excluded because of poor image quality, incomplete magnetic resonance images, or respiratory or metallic artifacts (Fig. 1). Therefore, 71 patients were enrolled in this study, including 33 male and 38 female patients.

All patients underwent T1WI, T2WI, 3D-STIR-SPACE, and T1WI enhancement sequences. The main clinical symptoms were lumps in the neck on the affected side, numbness in the hands, and swelling and weakness of the neck and upper limbs. Notably, eleven cases had no obvious symptoms of discomfort. Of the 71 patients, 46 were confirmed by surgery and pathology, nine were confirmed by tissue biopsy, and sixteen cases of schwannoma were clinically confirmed (Table 1).

**Table 1:** Demographic characteristics of study population

Characteristic	All Participants (*n* = 71)
Age, years	47.1 (8–79)
Sex	
Female	38 (53.52%)
Male	33 (46.48%)
Main clinical symptoms	
Lumps in the neck on the affected side	27 (38.02%)
Numbness in the hands	19 (26.76%)
Swelling and weakness of the neck and upper limbs	14 (19.71%)
No obvious symptoms	11 (15.49%)
Confirmation method	
Surgery and pathology	46 (64.79%)
Tissue biopsy	9 (12.68%)
Clinically confirmed	16 (22.53%)

Communication was initiated with patients and their families to inform them of the purpose and precautions of the examination, clarify the examination process, and eliminate the patient's psychological pressure. This study was approved by the hospital ethics committee, and all patients provided informed consent.

### MRI parameters

All patients were examined on a Siemens 3.0 T MRI scanner, and T1WI, T2WI, 3D-STIR-SPACE, and T1WI-enhanced sequences were conducted. For examination on the magnetic resonance scanner, the patient was placed in a supine position, raised his or her head and neck appropriately, and placed the arms by his or her side. In addition, the patient avoided deep breathing and swallowing throughout the process to minimize motion artifacts. The conventional scanning protocol was as follows: T1WI: TR = 650 ms, TE = 12 ms, field of view 40 cm × 40 cm, matrix 307 × 307, and slice thickness 4 mm; T2WI: TR = 3000 ms, TE = 101 ms, field of view 22 cm × 22 cm, matrix 314 × 314, and slice thickness 4 mm; 3D-STIR-SPAC: TR = 3000 ms, TE = 160 ms, field of view 42 cm × 42 cm, matrix 466 × 466, and slice thickness 3 mm;T1WI-enhanced sequences using dixon technique and the parameters are the same as T1WI(Table 2).

**Table 2:** Scanning sequence and main parameters of the brachial plexus

Scanning sequence	FOV (mm^2^)	Matrix	Slice thickness (mm)	TR/TE (ms)
T1WI	400 × 400	307 × 307	4.0	650/12
T2WI	220 × 220	314 × 314	4.0	3000/101
3D-STIR-SPACE	420 × 420	466 × 466	3.0	3000/160
T1WI + C	400 × 400	307 × 307	4.0	650/12

*FOV*  Field of view, *TR* Time of repetition, *TE*  Time of echo

All subjects underwent T1WI contrast enhancement images scanned 2 min 30 s after injection of the contrast agent Gd-DTPA at a dose of 0.2 mmol/kg injected intravenously with a high-pressure syringe. The coronary scanning range included the anterior and posterior edges of the vertebral canal, the upper edge of the second cervical vertebrae, and the upper edge of the second thoracic vertebrae. In addition, the axial scanning was based on the coronary position as a reference, and the distribution area of the fifth cervical nerve and the first thoracic nerve root on both sides was included in the scanning range.

### Image analysis

All images were analyzed by two attending physicians specializing in neuroimaging diagnosis. When there were differences of opinion, an agreement was reached after consultation. The tumor site, size, morphology, their relationship with the brachial plexus and surrounding structures, and the signal characteristics of all patients were summarized. The 3D-STIR-SPACE sequence was used to image the brachial plexus nerves, and the images were transmitted to the syngo.via VB40 (Siemens Healthcare, Erlangen, Germany) for processing. After MIP reconstruction of the brachial plexus nerves, soft tissues such as muscles were reduced to decrease interference with the anatomical positional relationship between the tumor and the nerves. After the tumor multiplanar reconstruction (MPR) was completed, the tumor range was sketched layer by layer, the brachial plexus nerve fused with the tumor image, and cVRT was used to render and obtain a three-dimensional model, which clearly showed the location and tissue structure of the brachial plexus nerves and the tumor in all directions. The three-dimensional image of the blood vessels was obtained in the same way by using T1WI-enhanced sequences. Finally, the three-dimensional images of the brachial plexus and tumor were fused with the three-dimensional images of the blood vessels. To better observe the relationship between the three, the transparency of the blood vessel image was adjusted to 65%.

## Results

Seventy-one patients (age, 8–79 years, mean, 47.1 years) were included in this study, and the brachial plexus nerve, surrounding tumor lesions, and vascular anatomy of all patients were well displayed through cVRT. Among the enrolled patients, there were 45 with schwannoma, fourteen with neurofibroma, ten with metastases, one with astrocytoma, and one with mediastinal malignant neuroblastoma.

### Site of the mass

Except for two cases of nerve sheath tumors that showed multiple lesions in the bilateral brachial plexus, the remainder of the patients had single foci in the brachial plexus. Thirty-five cases violated the left brachial plexus, and 33 violated the right brachial plexus. In addition, one case of astrocytoma was located in the left armpit, and one case of mediastinal malignant neuroblastoma was located on the right upper mediastinal membrane.

### Size and shape of the mass

The maximum diameter of all the lesions ranges from 1 to 10 cm (mean, 4.4 cm). Among them, the tumors of 53 patients that showed unilateral or bilateral growth along the brachial plexus nerve were fusiform, spherical, or multiple beaded, seven patients' tumors were circular, lobular, or irregular, and eleven patients' tumors had irregular morphology.

### Characteristics of mass signal

Forty-five patients with schwannoma showed a moderately uniform or uneven signal on T1WI and a high or mixed signal on T2WI. After enhancement, the signal was evenly or unevenly strengthened, and a low-signal area appeared in the middle of the mass when it was large. Fourteen cases of neurofibroma showed equally low signal on T1WI, equally high signal on T2WI, and uneven strengthening after enhancement. In addition, two of the ten cases of metastases showed equal signal on T1WI, high signal on T2WI, uniform signal, and uniform strengthening after enhancement. One case was an uneven and slightly lower signal on T1WI and a slightly higher mixed signal on T2WI that was unevenly strengthened after enhancement. One case of astrocytoma showed a mixed high signal on T1WI and a mixed high signal on T2WI, but no significant strengthening was seen after enhancement. In addition, one case of mediastinal malignant neuroblastoma had an equally low signal on T1WI and an equally high signal on T2WI. The signal was uneven, and it was significantly unevenly strengthened after enhancement.

### Relationship between the mass and the brachial plexus

There were 45 schwannoma cases, of which 26 showed multiple unilateral or bilateral tumors growing along the brachial plexus in a fusiform, spherical, or multiple-beaded pattern. Thirteen patients had spherical tumors encircling and compressing the brachial plexus, and six patients showed localized lesions encircling and infiltrating the brachial plexus. Among the fourteen neurofibroma cases, eleven had tumors growing along the brachial plexus in a fusiform or beaded pattern, or embedded in the brachial plexus. In addition, three patients had spherical tumors that encircled and compressed the brachial plexus. One case of astrocytoma showed diffuse tumor tissue and infiltrated the brachial plexus, and all the typical structures of the brachial plexus disappeared.

One case of mediastinal malignant neuroblastoma manifested as an abnormally high signal focal point of the upper right mediastinal membrane, with unclear boundaries and diffuse infiltration of the right brachial plexus. Of the ten cases of metastases, seven showed round, lobular, or irregular lesions with smooth boundaries, uniform signals, and pressure on the brachial plexus. In contrast, three showed diffuse infiltration of the soft tissues of the neck and the brachial plexus. The MRI characteristics of the brachial plexus-related neoplastic lesions are shown in Table 3. The table shows that the benign tumors were mostly round with smooth and clear edges, whereas the malignant tumors were mostly lobular with non-smooth edges.

**Table 3:** MRI findings of neoplasms involving the brachial plexus

Classifications	Schwannoma(*n* = 45)	Neurofibroma(*n* = 14)	Metastatic tumor(*n* = 10)	Astrocytoma(*n* = 1)	Malignant tumor of mediastinum(*n* = 1)
Size(cm)	3.6 ± 1.8	5.6 ± 2.9	5.7 ± 2.0	6.5	10.3
Form	Regular	Regular	Regular	Regular	Irregular
Boundary	Clear	Clear	Clear	Clear	Unclear
T1WI	Slightly hypointense	Slightly hypointense	Slightly hypointense	Hybrid high signal	Slightly hypointense
T2WI	Slightly hyperintense	Slightly hyperintense	Slightly hyperintense	Hybrid high signal	Slightly hyperintense
3D-STIR-SPACE	Slightly hyperintense	Slightly hyperintense	Slightly hyperintense	Hybrid high signal	Slightly hyperintense
Signal	Inhomogeneous	Inhomogeneous	Homogeneous	Inhomogeneous	Inhomogeneous
Envelope	45	No	No	No	No
Cystic degenerate hemorrhage	7/45	No	No	No	Yes
Enhancement pattern	Inhomogeneous	Inhomogeneous	Homogeneous	No enhancement	Inhomogeneous
Peripheral edema	No	No	No	No	Yes
Relationship with brachial plexus	Eccentric embedding	Wrapping	Push	Infiltration	Infiltration
Relationship with vascular	Push or soak	Push	Push	Push	Adhesion

The T1WI, T2WI, and 3D-STIR-SPACE sequences all use muscle signal as a reference

### The condition of the cVRT shows the tumor, the brachial plexus, and blood vessels

**Fig. 2:**
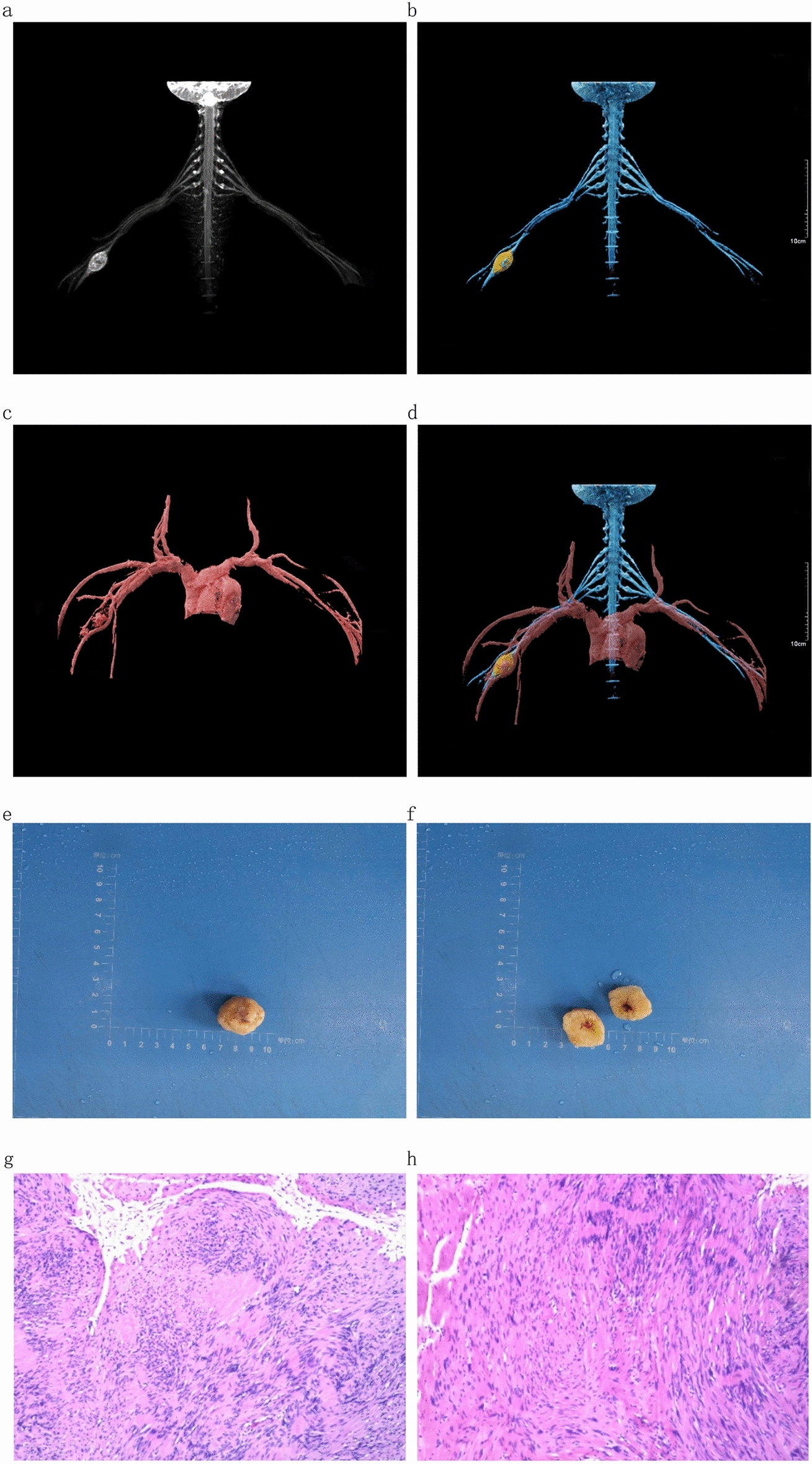
Case 1: 42-year-old female. a spindle mass at the beginning of the right axillary horizontal ulnar nerve can be seen in **a** and **b**, and the brachial plexus runs through the mass. **c,** **d** Right brachial artery trunk is compressed and shifted downward, and there are small arteries in the brachial artery branches to provide a blood supply for the tumor. The pathological results in **e** and **f** show that the section is gray and white, and it contains a bleeding remnant cavity, which is considered a schwannoma(**g** and **h**)

**Fig. 3:**
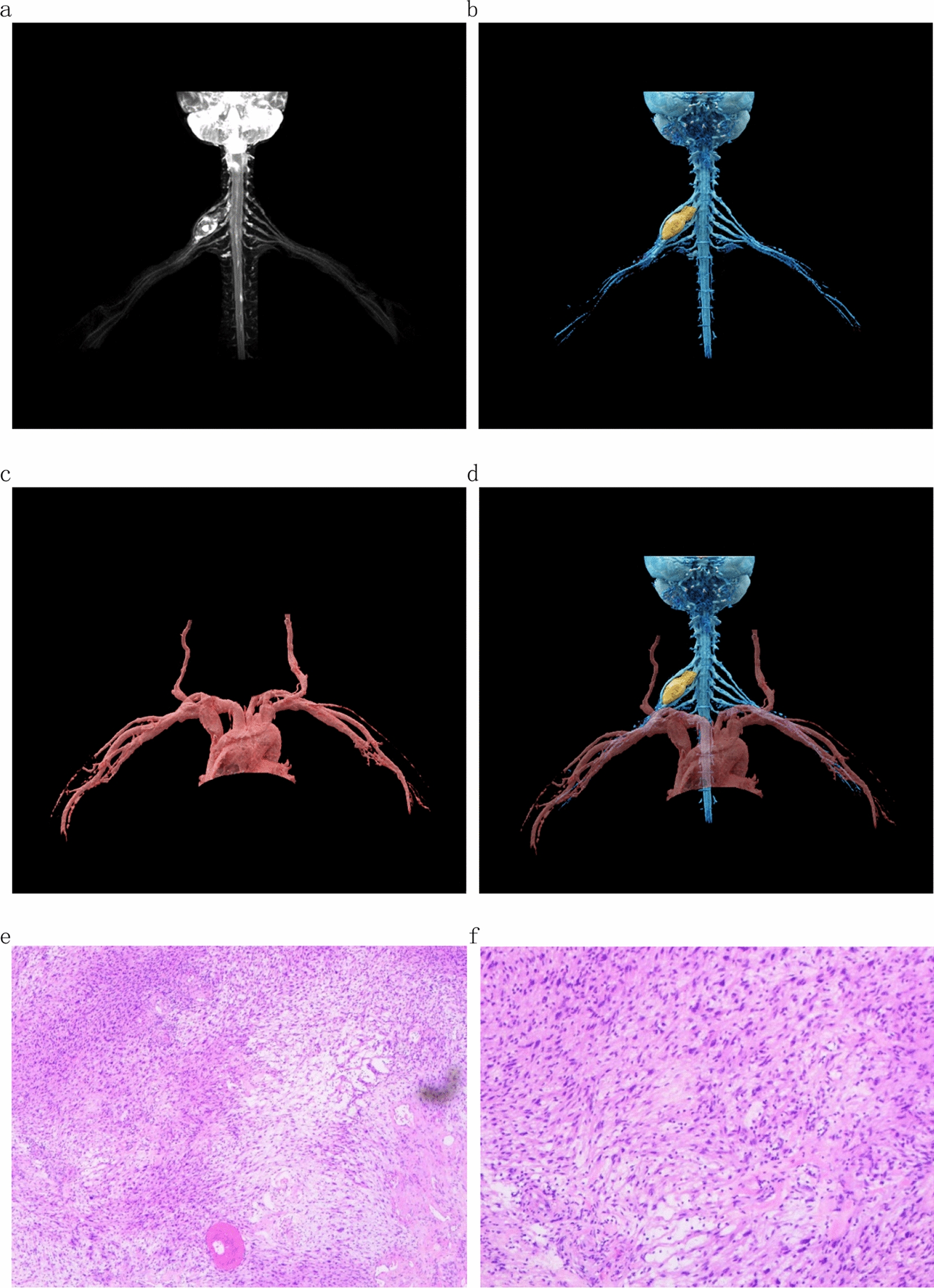
Case 2: 59-year-old female. **a,** **b** Spindle mass in the upper trunk of the right brachial plexus, closely related to the middle trunk, with the upper trunk and lower trunk surrounding the mass. **c**, **d** Right subclavian artery passes under the mass, and the boundary between the mass and the right subclavian artery is clear. The pathological results in **e** and **f** show a schwannoma

**Fig. 4:**
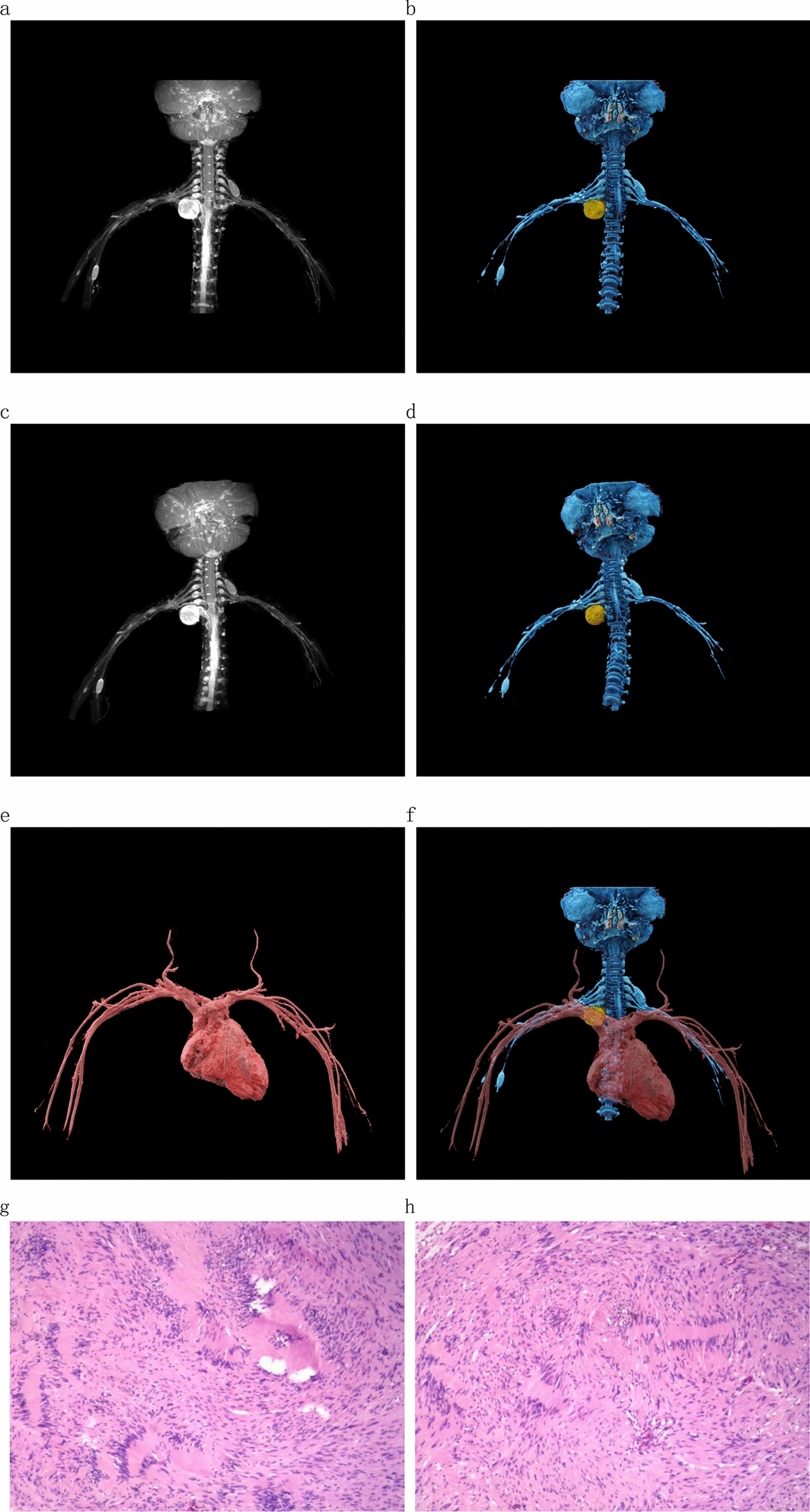
Case 3: 8-year-old female. **a**–**d** Spherical mass in the upper trunk of the right brachial plexus from different angles, closely related to the right brachial plexus, and the right cervical VIII nerve is compressed and uplifted. **e,** **f** right subclavian artery passes through the front of the mass, and the boundary between the mass and the right subclavian artery is clear. The pathological results in **g** and **h** show a schwannoma

The cVRT was statistically different from the T1WI 、T2WI and 3D-STIR-SPACE sequences in terms of clarifying the source of the tumor, the relationship between the tumor and the brachial plexus, and the tumor and blood vessels (*P* < 0.05, Table 4). The cVRT can better clarify the source of the tumor and the relationship among the tumor the brachial plexus and blood vessels than other sequences (Figs. 2, 3, 4).

**Table 4:** The cVRT and other sequences show the relationship among the tumor 、 the brachial plexus and blood vessels

Scanning sequence	Does or not the tumor originate from the brachial plexus nerve		Relationship between the tumor and the brachial plexus		Relationship between the tumor and the blood vessels	
	Determined	Not determined	Determined	Not determined	Determined	Not determined
T1WI	6	65	4	67	2	71
T2WI	8	53	6	65	3	71
3D-STIR-SPACE	60	11	58	13	0	71
T1WI + C	9	59	7	64	57	14
cVRT	68	3	65	6	63	8

The cVRT was compared with the T1WI,T2WI,3D-STIR-SPACE and T1WI + C sequences in terms of clarifying the source of the tumor, the relationship between the tumor and the brachial plexus, and the relationship between the tumor and blood vessels. *P* values are less than 0.05

## Discussion

The brachial plexus nerve is located superficially, and its anatomy is complex, making it susceptible to trauma, tumors, and other diseases. Therefore, accurate positioning and the qualitative diagnosis of brachial plexus neuropathy are essential for clinical treatment. The most effective clinical treatment method is surgical resection for patients with tumor lesions associated with the brachial plexus nerve and for brachial plexus nerve function. Imaging examination can locate and characterize brachial plexus nerve-related tumors, providing a solid clinical basis for surgical access and selection.

In 1993, Fler et al. [11] first described magnetic resonance neuroimaging (MRN). The application of this technology, including background inhibition diffusion-weighted imaging (DWIBS) [12] and 3D-STIR-SPACE [13, 14], has gradually been clinically recognized. The latter method has clear advantages. It can cover the entire brachial plexus nerve range, and accurately evaluate the deformation, compression, and interruption of post-ganglion nerve fibers [15]. Using the 3D-STIR-SPACE sequence with enhanced scanning on a 3.0 T magnetic resonance scanner can clearly and intuitively demonstrate the three-dimensional display of the composition and continuity of the bilateral brachial plexus, accurately locate and diagnose tumors and other diseases involving the brachial plexus, determine the site and degree of damage to each nerve, and help clinicians choose appropriate treatment options and surgical methods [16].

To be able to clearly and vividly show the relationship between the tumor placeholder and brachial plexus nerve, the studies [17] using 3D-MRI technology to examine brachial plexus neuropathy caused by tumor compression have been conducted. However, this technology is not yet able to effectively distinguish the surrounding related tumors from the brachial plexus nerve wrapping and infiltration, so new imaging or post-processing techniques are needed to provide a practical imaging reference for clinical use.

The cVRT is a movie-level real-time rendering technology and an accurate physical simulation technology based on the interaction of light and matter. Multiple light sources are used to produce various interactions between light and human tissues (e.g., reflection, refraction, primary scattering, and secondary scattering). Depth and morphological perception have been enriched and enhanced, forming a more realistic shadow, which accurately shows the anatomical level of soft tissues and blood vessels. At the same time, the three-dimensional anatomical effect tends to be more realistic, providing more detailed and accurate information for clinical practice. Guo B et al. [18] has applied Hyperrealistic Rendering to display Type II Endoleak, and the three-dimensional display is more intuitive and realistic, providing more accurate guidance and suggestions for clinical preoperative evaluation, and improving confidence in the surgery.

Tumors related to the brachial plexus nerve area are usually completely removed by surgery. A comprehensive assessment is required before any operation or the brachial plexus nerve will be easily damaged during surgery [19, 20]. Therefore, this study tried to use cVRT based on 3D-SPACE-STIR brachial plexus imaging, combined with enhanced magnetic resonance imaging, to obtain a three-dimensional fusion image of the tumor, brachial plexus nerve and blood vessels after processing. Different color levels were used to distinguish the three tissues. In this model, the spatial and positional relationship between the tumor, and the brachial plexus nerve and blood vessels were well-reflected.

Of the 71 patients in this study, 46 were treated surgically and 25 conservatively. In Case 1, a 42-year-old female, the three-dimensional fusion image obtained after treatment with cVRT technology showed that the brachial plexus nerve traveled through the middle of the tumor, which was closely associated with the brachial plexus nerve. In addition, the trunk of the right brachial artery was displaced downward under pressure, and the branches of the brachial artery had small arteries that supplied blood to the tumor. The operation was difficult. Gentle manipulation was done during the operation to separate the branches of the brachial artery around the tumor to avoid damage to the brachial plexus nerve. The tumor was cut out slowly, producing minimal bleeding, consistent with the results of the imaging examination. Then, the tumor was completely removed. The patient recovered well after the operation.

In Case 2, a 59-year-old female, the image after treatment with cVRT technology showed that the tumor was closely related to the sixth cervical nerve, and the fifth and seventh cervical nerves surrounded the tumor. The right subclavian artery passed under the tumor but the boundary was clear. The subclavian artery and the fifth and seventh cervical nerves were gently separated during the operation. The tumor was slowly incised and carefully exfoliated after exposing it to avoid damage to the sixth cervical nerve. The patient recovered well after the operation.

In Case 3, an 8-year-old female, the tumor under the eight cervical nerve on the right side could be seen after treatment with cVRT technology. It was closely related to the right brachial plexus. The eighth cervical nerve on the right was compressed and raised, and the right subclavian artery passed in front of the mass. After observation of 360° rotation, it was found that the boundary between the eighth cervical nerve and the subclavian artery on the right was clear. During the operation, the right eighth cervical nerve and the right subclavian artery were carefully separated, and the tumor was completely removed. The patient recovered well after the operation.

In clinical practice, visual research has been conducted on the diagnosis of brachial plexus nerve injury, and related research has also been conducted on the timing and method selection of brachial plexus nerve injury, and preliminary results have been achieved. We have also continuously tried clinical applications and found that when tumor metastases invade the brachial plexus nerve and surrounding blood vessels, cVRT vascular nerve fusion technology can also show lesions to a great extent. In addition, it can also play a guiding role in the treating the brachial plexus nerve and surrounding lesions caused by tumor compression.

## Conclusion

The cVRT showed the anatomical structure of the brachial plexus with surrounding tumor lesions and blood vessels in three dimensions, providing more accurate and realistic image information for clinical use.

## Data Availability

All data generated or analysed during this study are included in this published article.

## References

[1] Tharin BD, Kini JA, York GE, Ritter JL (2014). Brachial plexopathy: a review of traumatic and nontraumatic causes. AJR Am J Roentgenol.

[2] Kim DH, Murovic JA, Tiel RL, Moes G, Kline DG (2005). A series of 397 peripheral neural sheath tumors: 30-year experience at Louisiana State University Health Sciences Center. J Neurosurg.

[3] Kehoe NJ, Reid RP, Semple JC (1995). Solitary benign peripheral-nerve tumours. Review of 32 years’ experience. J Bone Joint Surg Br.

[4] Siqueira MG, Martins RS, Teixeira MJ (2009). Management of brachial plexus region tumours and tumour-like conditions: relevant diagnostic and surgical features in a consecutive series of eighteen patients. Acta Neurochir.

[5] Jia X, Yang J, Chen L, Yu C, Kondo T (2016). Primary brachial plexus tumors: clinical experiences of 143 cases. Clin Neurol Neurosurg.

[6] Somashekar D, Yang LJ, Ibrahim M, Parmar HA (2014). High-resolution MRI evaluation of neonatal brachial plexus palsy: a promising alternative to traditional CT myelography. AJNR Am J Neuroradiol.

[7] Upadhyaya V, Upadhyaya DN, Kumar A, Pandey AK, Gujral R, Singh AK (2015). Magnetic resonance neurography of the brachial plexus. Indian J Plast Surg.

[8] Wade RG, Takwoingi Y, Wormald JCR, Ridgway JP, Tanner S, Rankine JJ, Bourke G (2019). MRI for detecting root avulsions in traumatic adult brachial plexus injuries: a systematic review and meta-analysis of diagnostic accuracy. Radiology.

[9] Amrami KK, Felmlee JP, Spinner RJ (2008). MRI of peripheral nerves. Neurosurg Clin N Am.

[10] Johnson PT, Schneider R, Lugo-Fagundo C, Johnson MB, Fishman EK (2017). MDCT angiography With 3D rendering: a novel cinematic rendering algorithm for enhanced anatomic detail. AJR Am J Roentgenol.

[11] Filler AG, Howe FA, Hayes CE, Kliot M, Winn HR, Bell BA, Griffiths JR, Tsuruda JS (1993). Magnetic resonance neurography. Lancet.

[12] Takahara T, Imai Y, Yamashita T, Yasuda S, Nasu S, Van Cauteren M (2004). Diffusion weighted whole body imaging with background body signal suppression (DWIBS): technical improvement using free breathing, STIR and high resolution 3D display. Radiat Med.

[13] Viallon M, Vargas MI, Jlassi H, Lövblad KO, Delavelle J (2008). High-resolution and functional magnetic resonance imaging of the brachial plexus using an isotropic 3D T2 STIR (Short Term Inversion Recovery) SPACE sequence and diffusion tensor imaging. Eur Radiol.

[14] Zhang L, Xiao T, Yu Q, Li Y, Shen F, Li W (2018). Clinical value and diagnostic accuracy of 3.0T multi-parameter magnetic resonance imaging in traumatic brachial plexus injury. Med Sci Monit.

[15] Vargas MI, Viallon M, Nguyen D, Beaulieu JY, Delavelle J, Becker M (2010). New approaches in imaging of the brachial plexus. Eur J Radiol.

[16] Tagliafico A, Succio G, Neumaier CE, Baio G, Serafini G, Ghidara M, Calabrese M, Martinoli C (2012). Brachial plexus assessment with three-dimensional isotropic resolution fast spin echo MRI: comparison with conventional MRI at 3.0 T. Br J Radiol.

[17] Chhabra A, Thawait GK, Soldatos T, Thakkar RS, Del Grande F, Chalian M, Carrino JA (2013). High-resolution 3T MR neurography of the brachial plexus and its branches, with emphasis on 3D imaging. AJNR Am J Neuroradiol.

[18] Guo B, Peng L (2023). Hyperrealistic rendering of type II endoleak. Radiology.

[19] Lee HJ, Kim JH, Rhee SH, Gong HS, Baek GH (2014). Is surgery for brachial plexus schwannomas safe and effective?. Clin Orthop Relat Res.

[20] Martin E, Senders JT, DiRisio AC, Smith TR, Broekman MLD (2018). Timing of surgery in traumatic brachial plexus injury: a systematic review. J Neurosurg.

